# The Phosphodiesterase-5 Inhibitor Vardenafil Is a Potent Inhibitor of ABCB1/P-Glycoprotein Transporter

**DOI:** 10.1371/journal.pone.0019329

**Published:** 2011-04-28

**Authors:** Pei-Rong Ding, Amit K. Tiwari, Shinobu Ohnuma, Jeferson W. K. K. Lee, Xin An, Chun-Ling Dai, Qi-Si Lu, Satyakam Singh, Dong-Hua Yang, Tanaji T. Talele, Suresh V. Ambudkar, Zhe-Sheng Chen

**Affiliations:** 1 Department of Pharmaceutical Sciences, College of Pharmacy and Allied Health Professions, St. John's University, Jamaica, New York, United States of America; 2 State Key Laboratory of Oncology in South China, Cancer Center, Sun Yat-Sen University, Guangzhou, China; 3 Department of Colorectal Surgery, Cancer Center, Sun Yat-Sen University, Guangzhou, China; 4 Laboratory of Cell Biology, Center for Cancer Research, National Cancer Institute, National Institutes of Health, Bethesda, Maryland, United States of America; 5 Department of Medical Oncology, Cancer Center, Sun Yat-Sen University, Guangzhou, China; 6 Biosample Repository, Fox Chase Cancer Center, Philadelphia, Pennsylvania, United States of America; IIT Research Institute, United States of America

## Abstract

One of the major causes of chemotherapy failure in cancer treatment is multidrug resistance (MDR) which is mediated by the ABCB1/P-glycoprotein. Previously, through the use of an extensive screening process, we found that vardenafil, a phosphodiesterase 5 (PDE-5) inhibitor significantly reverses MDR in ABCB1 overexpressing cancer cells, and its efficacy was greater than that of tadalafil, another PDE-5 inhibitor. The present study was designed to determine the reversal mechanisms of vardenafil and tadalafil on ABC transporters-mediated MDR. Vardenafil or tadalafil alone, at concentrations up to 20 µM, had no significant toxic effects on any of the cell lines used in this study, regardless of their membrane transporter status. However, vardenafil when used in combination with anticancer substrates of ABCB1, significantly potentiated their cytotoxicity in ABCB1 overexpressing cells in a concentration-dependent manner, and this effect was greater than that of tadalafil. The sensitivity of the parenteral cell lines to cytotoxic anticancer drugs was not significantly altered by vardenafil. The differential effects of vardenafil and tadalafil appear to be specific for the ABCB1 transporter as both vardenafil and tadalafil had no significant effect on the reversal of drug resistance conferred by ABCC1 (MRP1) and ABCG2 (BCRP) transporters. Vardenafil significantly increased the intracellular accumulation of [^3^H]-paclitaxel in the ABCB1 overexpressing KB-C2 cells. In addition, vardenafil significantly stimulated the ATPase activity of ABCB1 and inhibited the photolabeling of ABCB1 with [^125^I]-IAAP. Furthermore, Western blot analysis indicated the incubation of cells with either vardenafil or tadalafil for 72 h did not alter ABCB1 protein expression. Overall, our results suggest that vardenafil reverses ABCB1-mediated MDR by directly blocking the drug efflux function of ABCB1.

## Introduction

The resistance of tumor cells to a variety of structurally and mechanistically unrelated cytotoxic drugs, also known as multidrug resistance (MDR), is one of the major obstacles in the successful treatment of cancer [1]. It is estimated that approximately 500,000 new cases of cancer each year exhibit the drug resistant phenotype [2]. One of the known causes of MDR is overexpression of the ATP-binding cassette (ABC) transporters, such as P-glycoprotein (ABCB1/P-gp), multidrug resistance proteins (ABCCs/MRPs) and breast cancer resistant protein (ABCG2/BCRP). These transporters actively efflux a variety of structurally and functionally diverse chemotherapeutic drugs out of cancer cells, thereby reducing the intracellular drug accumulation, increasing the likelihood of decreased cytotoxic and thus unsuccessful treatment [3], [4], [5], [6]. Currently, 48 distinct ABC transporters have been identified in the human genome, and these can further divided into seven subfamilies (A–G) based on sequence similarities [3]. Among these transporters, the ABCB1 transporter is the most important mediator of MDR [7], [8], and is responsible for chemotherapeutic drug resistance to a variety of drug, including vinca alkaloids, anthracyclines, epipodophyllotoxins and taxanes [9]. The overexpression of ABCB1 occurs in 40–50% of cancer patients [10], and is associated with a poor clinical outcome [11], [12]. Based on these findings, a number of studies have attempted to selectively inhibit ABCB1 activity as a strategy to reverse MDR in cancer chemotherapy. Indeed, in the past 30 years, significant efforts have been made to design and test specific ABCB1 inhibitors and this has resulted in the development of three generations of ABCB1 inhibitors. However, currently, none of the compounds in the three generations have been approved for clinical use. The first-generation ABCB1 inhibitors, including verapamil, quinine, and cyclosporin A lacked selectivity and produced undesirable adverse effects at plasma concentrations necessary to inhibit ABCB1 [13]. The second-generation ABCB1 inhibitors, such as valspodar/PSC-833 and biricodar/VX-710, had improved tolerability compared to the first-generation compounds. However, they produced unpredictable interactions with other transport proteins and inhibited CYP3A4, one of the major chemotherapeutic drug metabolizing enzymes, thereby reducing the the clearance and metabolism of chemotherapeutic drugs [14]. The third-generation inhibitors were more selective for the ABCB1 transporters in ongoing clinical trials. Nonetheless, some of these compounds produced significant adverse effects and had an unfavorable pharmacokinetic profile, including poor solubility as well as reducing the clearance of clinically used anticancer drugs [15]. Recent results from our laboratory and others indicate that several tyrosine kinase inhibitors (TKIs), including imatinib [16], nilotinib [17], lapatinib [18], and erlotinib [19], can reverse MDR to antineoplastic drugs mediated by ABC-transporters. However, the reversal potential of these TKIs have not been determined in clinical trials. Consequently, it is necessary to develop more efficacious, non-toxic and less expensive compounds to reverse MDR in cancer cells. In the course of our search for compounds that reverse MDR, we found that vardenafil and tadalafil, two phosphodiesterase type-5 (PDE-5) inhibitors clinically used in the treatment of male erectile dysfunction, significantly reversed ABCB1-mediated MDR. In the present study, we conducted experiments to ascertain the reversal mechanism of vardenafil and tadalafil in ABCB1 overexpressing cancer cells. In addition, we also examined their effect on other major ABC drug transporters such as MRP1 and BCRP.

## Materials and Methods

### Reagents

Vardenafil and tadalafil were purchased from Toronto Research Chemicals Inc. (Ontario, Canada). [^3^H]-paclitaxel (37.9 Ci/mmol) was purchased from Moravek Biochemicals Inc (Brea, CA). [^125^I]-Iodoarylazidoprazosin (IAAP) (2,200 Ci/mmol) was obtained from Perkin Elmer Life Sciences (Boston, MA). Monoclonal antibody C-219 (against ABCB1) was acquired from Signet Laboratories Inc. (Dedham, MA). Anti-glyceraldehyde-3-phosphate dehydrogenase (GAPDH) monoclonal antibody (14C10) was obtained from Cell Signaling Technology, Inc. (Danvers, MA). Fumitremorgin C (FTC) was synthesized by Thomas McCloud Developmental Therapeutics Program, Natural Products Extraction Laboratory, NCI, NIH (Bethesda, MD). ONO1078 was a gift from Dr. Akiyama (Kagoshima University, Japan). Paclitaxel, vincrinstine (VCR), colchicine, 7-ethyl-10-hydroxy-20 (S)-camptothecin (SN-38), verapamil, dimethyl sulfoxide (DMSO), 1-(4,5-dimethylthiazol-2-yl)-3,5-diphenylformazan (MTT) and all other chemicals were purchased from Sigma Chemical Co. (St. Louis, MO).

### Cell lines and cell culture

The ABCB1/P-gp-overexpressing drug-resistant cell line KB-C2 was established in a cell culture medium by a step-wise selection of the parental human epidermoid carcinoma cell line KB-3-1 using colchicine at concentrations up to 2 µg/ml [20]. The KB-C2 and KB-3-1 were kindly provided by Dr Shin-ichi Akiyama (Kagoshima University, Japan). HEK293-pcDNA3.1 and wild-type HEK/ABCG2 transfected cells were established by selection with G418 after transfecting HEK293 with either empty pcDNA3.1 vector or pcDNA3.1 vector containing full length of ABCG2 coding arginine (R) at 482 amino acid position, respectively, and were then cultured in a medium with 2 mg/ml of G418 [21]. Similarly, the HEK/MRP1 (ABCC1) and HEK/ABCB1 cells were generated by transfecting the HEK293 with the MRP1 expression vector and the ABCB1 expressing vector, respectively [22]. All of the cell lines were grown in Dulbecco's modified Eagle's medium (DMEM) supplemented with 10% bovine serum, 100 units/ml penicillin, and 100 mg/ml streptomycin in a humidified incubator containing 5% CO_2_ at 37°C.

### Cell cytotoxicity by MTT assay

The MTT assay was used to assess cytotoxicity. The cultured cells were harvested with trypsin and resuspended in a final concentration of 4×10^3^ cells/well for KB-3-1, 7.5×10^3^ cells/well for KB-C2 and 8×10^3^ for all of the other cell lines used in this study. Cells were seeded evenly in 96 well multiplates. In the reversal experiments, different concentrations of chemotherapeutic drugs (20 µl/well) were added into designated wells after 1 h with or without exposure to potential reversal compounds vardenafil, tadalafil, verapamil, ONO-1078, or FTC (20 µl/well). After 68 h of incubation, 20 µl of the MTT solution (4 mg/ml) was added to each well, and the plate was further incubated for 4 h at 37°C, allowing viable cells to convert the yellow-colored MTT into dark-blue formazan crystals. Subsequently, the medium was discarded, and 100 µl of DMSO was added into each well to dissolve the formazan crystals generating purple color. The absorbance was determined at 570 nm by an OPSYS Microplate Reader from DYNEX Technologies, Inc. (Chantilly, VA). The degree of resistance was calculated by dividing the IC_50_ (concentrations required to inhibit growth by 50%) for the resistant cells by that of the parental sensitive cells. The degree of the reversal of MDR was calculated by dividing the IC_50_ for cells with the anticancer drug in the presence or absence of reversal agents by that of parental cells with anticancer drugs obtained in the absence of reversal agent. The IC_50_ values were calculated from survival curves using the Bliss method.

### [^3^H]-paclitaxel accumulation and efflux

The intracellular accumulation of [^3^H]-paclitaxel was measured as previously described [23]. Briefly, confluent cells in 24-well plates were preincubated with or without the reversing agents for 1 h at 37°C. Intracellular paclitaxel accumulation was measured by incubating cells with 0.1 µM [^3^H]-paclitaxel for 2 h in the presence or absence of the reversing agents at 37°C. The cells were washed three times with ice-cold PBS, then suspended in fresh medium with or without reversing agents at 37°C. Aliquots of the extracellular medium (40 µl) were collected at various time points (0, 60, 120 min) and finally the cells were collected and lysed in 10 mM lysis buffer (pH 7.4, containing 1% Triton X-100 and 0.2% SDS). Each sample was placed in scintillation fluid and the radioactivity was measured in a Packard TRI-CARB1 1900CA liquid scintillation analyzer from Packard Instrument Company, Inc. (Downers Grove, IL).

### Western blot and immunofluorescence analysis

To determine the effect of vardenafil or tadalafil on the expression of ABCB1, KB-C2 cells were incubated with 10 µM vardenafil or tadalafil for 0, 36 and 72 h. Following incubation, the cells were harvested and rinsed twice with ice-cold PBS and total cell lysates were collected with cell lysis buffer (1× PBS, 1% Nonidet P-40, 0.5% sodium deoxycholate, 0.1% SDS, 100 µg/ml phenylmethylsulfonyl fluoride, 10 µg/ml aprotinin, 10 µg/ml leupeptin) for 30 min with gentle rocking and clarified by centrifugation at 12,000 rpm for 10 min at 4°C. Equal amounts (100 µg of protein) of cell lysates were resolved by sodium dodecyl sulfate polyacrylamide gel electrophoresis (SDS-PAGE) and electrophoretically transferred onto polyvinylidene fluoride (PVDF) membranes. After incubation in a blocking solution containing 5% non-fat milk in TBST buffer (10 mM Tris-HCL (pH 8.0), 150 mM NaCl, and 0.1% Tween 20) for 1 h at room temperature, membranes were immunoblotted overnight with primary monoclonal antibodies C219 (1∶200) against ABCB1 or 14C10 (1∶200) against GAPDH at 4°C. Subsequently, the membranes were washed three times for 15 min with TBST buffer and incubated at room temperature for 2 h with HRP-conjugated secondary antibody at 1∶1000 dilutions. The membranes underwent three additional washes for 15 min with TBST buffer and the protein-antibody complex were visualized by the enhanced Phototope TM-HRP Detection Kit (Cell Signaling, USA) and exposed to Kodak medical X-ray processor (Kodak, USA) [18]. For the immunofluorescence analysis, cells (2×10^3^) were seeded in 24 well plates and vardenafil or tadalafil at 10 µM were added into the wells after 12 h of incubation at 37°C in a humidified atmosphere of 5% CO2. After incubation for 72 h of incubation, cells were washed with PBS and fixed with 4% paraformaldehyde for 15 min at room temperature and then rinsed with PBS three times. A monoclonal antibody C219 against ABCB1 (1∶500) (Signet Laboratories Inc., Dedham, MA) was added and incubated overnight and Alexa flour 488 goat antimouse IgG (1∶1000, Molecular Probe, Carlsbad, CA) was added and cultured for 1 h. Propidium iodide was used for nuclear counterstaining.

### ATPase assay of ABCB1

The Vi-sensitive ATPase activity of ABCB1 in membrane vesicles of High-Five insect cells was measured as previously described [24]. Briefly, the membrane vesicles (10 µg of protein) were incubated in ATPase assay buffer (50 mM MES [pH 6.8], 50 mM KCl, 5 mM sodium azide, 2 mM EGTA, 2 mM dithiothreitol, 1 mM ouabain and 10 mM MgCl_2_) with or without 0.3 mM orthovanadate (freshly prepared) at 37°C for 5 min, then incubated with different concentrations of drug at 37°C for 3 min. The ATPase reaction was started by the addition of 5 mM ATP, and the total volume was 0.1 ml. After incubation at 37°C for 20 min, the reactions were stopped by the addition of 0.1 ml of 5% SDS solution and vortexed and kept at room temperature. The liberated P_i_ was measured as previously described [24].

### Photoaffinity labeling of ABCB1 with [^125^I]-IAAP

The photoaffinity labeling of ABCB1 with [^125^I]-IAAP was performed as previously described [25]. The membrane vesicles from High-Five insect cells expressing ABCB1 (50 µg of protein) were incubated at room temperature with different concentrations of drugs in the ATPase assay buffer with [^125^I]-IAAP (7 nM) for 5 min under subdued light. The samples were photo cross-linked by using a 365 nm UV light source for 10 min at room temperature. After the samples were placed in an SDS-PAGE in a 7% Tris-acetate NuPAGE gel, the gels were dried and exposed to Bio-Max MR film (Eastman Kodak Co., NY, USA) at −70°C for 8–12 h. The radioactivity incorporated into the ABCB1 band was quantified using the STORM 860 PhosphorImager system and ImageQuaNT (Molecular Dynamics, CA).

### Ligand-ABCB1 structure preparation

Vardenafil, tadalafil (modeled as *R*,*R* isomer) and IAAP were constructed using the fragment dictionary of Maestro 9.0 and the energy minimized by Macromodel program v9.7 (Schrödinger, Inc., New York, NY, 2009) using the OPLSAA force field [26] with the steepest descent followed by truncated Newton conjugate gradient protocol. Partial atomic charges were computed using the OPLS-AA force field. The low-energy 3D structures of vardenafil, tadalafil and IAAP were generated with the following parameters present in LigPrep v2.3: different protonation states at physiological pH, all possible tautomers and ring conformations.

### Protein structure preparation

The X-ray crystal structure of ABCB1 in the apoprotein state (PDB ID: 3G5U) and in complex with inhibitors QZ59-*RRR* (PDB ID: 3G6O) and QZ59-*SSS* (PDB ID: 3G61) was obtained from the RCSB Protein Data Bank and were used to build the homology model of human ABCB1 [27]. The homology modeling was conducted using the default parameters of Prime v2.1 as implemented in Maestro 9.0. The input file for the amino acid sequence of human ABCB1 in the Prime structure prediction application was obtained as fasta file (uniprot accession number P08183.3) extracted from http://www.uniprot.org. The co-crystal structures of ABCB1 from the mouse model in complex with QZ59-*RRR* and QZ59-*SSS* inhibitors were used as templates for modeling site-1 and site-2, respectively; while apoprotein-ABCB1 was used as a template for modeling the site-3 and site-4. The resultant alignment of human ABCB1 and mouse ABCB1 sequences produced 87% sequence identity and 93% similarity. Based on the resultant alignment that was constructed using default parameters, the side chains were optimized and residues were minimized. The initial structure thus obtained was refined by means of default parameters mentioned in protein preparation facility implemented in Maestro v9.0 and Impact program v5.5 (Schrödinger, Inc., New York, NY, 2009), in which the protonation states of residues were adjusted to the dominant ionic forms at pH 7.4. Refined human ABCB1 homology model was further used to generate four different receptor grids by selecting QZ59-*RRR* (site-1) and QZ59-*SSS* (site-2) bound ligands, all amino acid residues known to contribute to verapamil binding (site-3) and two residues known to be common to three previous sites (site-4) as shown in Table S1.

### Docking protocol

The docking calculations were performed using the “Extra Precision” (XP) mode of Glide program v5.5 (Schrödinger, Inc., New York, NY, 2009) and the default parameters. The top scoring pose-ABCB1 complex was then subjected to energy minimization using Macromodel program v9.7 using the OPLS-AA force field [26] and used for graphical analysis. All computations were carried out on a Dell Precision 470 n dual processor with the Linux OS (Red Hat Enterprise WS 4.0).

### Statistical Analysis

All experiments were repeated at least three times and the differences were determined by using the Student's t-test. The statistical significance was determined at *p*<0.05.

## Results

### Vardenafil significantly enhances the drug sensitivity of ABCB1 overexpressing cancer cells but does not alter the drug sensitivity in ABCC1 and ABCG2 overexpressing cells

Prior to determining if MDR could be reversed by vardenafil or tadalafil, we first determined their cytotoxic effects in different cell lines using the MTT assay. The results indicated that both vardenafil and tadalafil did not inhibit the growth of any of the cell lines used in this study at concentrations up to 20 µM (data not shown). We then determined the effect of vardenafil or tadalafil on the sensitivity of anticancer drugs in ABCB1-, ABCC1-, and ABCG2-overexpressing MDR cells. As shown in Table 1, vardenafil at 5 and 10 µM, produced a concentration-dependent increase in the cytotoxicity of colchicine and paclitaxel in ABCB1-overexpressing KB-C2 cells. Similarly, vardenafil increased the sensitivity of ABCB1 transfected HEK/ABCB1 cells to vincristine and paclitaxel (Table 2). In contrast, vardenafil did not significantly alter the cytotoxicity of the tested drugs in the parental sensitive KB-3-1 cells (Table 1). In addition, vardenafil did not reverse MDR induced by cells expressing ABCC1 and ABCG2 (Table S2) or significantly alter the IC_50_ values of cisplatin, which is not a substrate of ABCB1 (Table 1 and 2). We also determined if MDR could be reversed by tadalafil, another PDE-5 inhibitor. The results indicated that tadalafil, at concentrations of 5 or 10 µM also significantly enhanced the colchicine and paclitaxel sensitivity in KB-C2 cells and the vincristine and paclitaxel in HEK/ABCB1 cells. However, the fold reversal was significantly less than that of vardenafil. (Table 1 and 2).

**Table 1:** The effect of vardenafil and tadalafil on the reversal of ABCB1-mediated resistance to colchicine, paclitaxel and cisplatin in drug selected cell line.

Compounds	IC50 ± SD (µM) (fold reversal)	
	KB-3-1	KB-C2 (ABCB1)
Colchicine	0.0063±0.0013 (1.00)	2.9017±0.6127 (1.00)
+Vardenafil 5 µM	0.0070±0.0014 (0.90)	0.1053±0.0215** (27.6)
+Vardenafil 10 µM	0.0068±0.0012 (0.93)	0.0157±0.0063** (184.8)
+Tadalafil 5 µM	0.0073±0.0009 (0.86)	0.6923±0.1518** (4.19)
+Tadalafil 10 µM	0.0071±0.0015 (0.89)	0.4547±0.1033** (6.38)
+Verapamil 10 µM	0.0064±0.0011 (0.98)	0.0347±0.0071** (83.6)
Paclitaxel	0.0066±0.0016 (1.00)	0.7354±0.0141 (1.00)
+Vardenafil 5 µM	0.0074±0.0015 (0.89)	0.0281±0.0067** (26.2)
+Vardenafil 10 µM	0.0072±0.0017 (0.92)	0.0136±0.0025** (54.1)
+Tadalafil 5 µM	0.0071±0.0020 (0.93)	0.2911±0.0669** (2.53)
+Tadalafil 10 µM	0.0065±0.0028 (1.02)	0.1641±0.0311** (4.48)
+Verapamil 10 µM	0.0060±0.0009 (1.10)	0.0132±0.0035** (55.7)
Cisplatin	1.9032±0.0709 (1.00)	1.7877±0.2171 (1.00)
+Vardenafil 5 µM	2.1316±0.3653 (0.89)	2.0555±0.7811 (0.87)
+Vardenafil 10 µM	2.0364±0.6313 (0.93)	1.8651±0.5409 (0.96)
+Tadalafil 5 µM	1.9899±0.4975 (0.96)	1.7700±0.7257 (1.01)
+Tadalafil 10 µM	1.7890±0.6083 (1.06)	1.9221±0.9034 (0.93)
+Verapamil 10 µM	1.8703±0.2330 (1.02)	1.7890±0.6472 (1.00)

Cell survival was determined by MTT assay as described in “Materials and Methods”. Data are expressed as the means ± SD of at least three independent experiments performed in triplicate. The magnitude of the fold-reversal of MDR fold reversal (values given in parentheses) was calculated by dividing the IC_50_ for cells with the anticancer drug in the absence of inhibitor by that obtained in the presence of inhibitor.

**represents *P*<0.01, for values versus that obtained in the absence of inhibitor.

**Table 2:** The effect of vardenafil and tadalafil on the reversal of ABCB1-mediated resistance to vincristine, paclitaxel and cisplatin in ABCB1 transfected cell line.

Compounds	IC50 ± SD (µM) (fold reversal)	
	HEK293/pcDNA3.1	HEK/ABCB1 (ABCB1)
Vincristine	11.55±1.59 (1.00)	169.84±12.93 (1.00)
+Vardenafil 5 µM	13.13±0.93 (0.88)	41.41±6.12** (4.10)
+Vardenafil 10 µM	9.72±2.13 (1.19)	14.64±2.36** (11.60)
+Tadalafil 5 µM	10.02±1.24 (1.15)	112.94±5.04 (1.50)
+Tadalafil 10 µM	8.57±0.62 (1.35)	69.27±3.48* (2.45)
+Verapamil 10 µM	8.23±1.11 (1.40)	18.21±4.82** (9.33)
Paclitaxel	23.73±5.21 (1.00)	219.14±13.16 (1.00)
+Vardenafil 5 µM	21.85±2.04 (1.09)	56.83±7.74** (3.86)
+Vardenafil 10 µM	20.09±3.06 (1.18)	25.23±4.17** (8.69)
+Tadalafil 5 µM	24.04±2.95 (0.99)	159.78±11.52 (1.37)
+Tadalafil 10 µM	19.64±3.17 (1.21)	96.92±4.83* (2.26)
+Verapamil 10 µM	20.17±2.52 (1.18)	31.03±2.19** (7.06)
Cisplatin	890.32±33.92 (1.00)	850.84±82.53 (1.00)
+Vardenafil 5 µM	839.03±48.37 (1.06)	893.19±32.60 (0.95)
+Vardenafil 10 µM	892.44±19.26 (1.00)	782.82±59.31 (1.09)
+Tadalafil 5 µM	901.29±67.23 (0.99)	909.06±98.44 (0.94)
+Tadalafil 10 µM	792.85±92.60 (1.12)	783.60±84.20 (1.09)
+Verapamil 10 µM	853.62±61.04 (1.04)	725.71±48.56 (1.17)

Cell survival was determined by MTT assay as described in “Materials and Methods”. Data are expressed as the means ± SD of at least three independent experiments performed in triplicate. The fold-The magnitude of the MDR fold reversalreversal of MDR ((values given in parentheses) was calculated by dividing the IC_50_ for cells with the anticancer drug in the absence of inhibitor by that obtained in the presence of inhibitor.

**represents *P*<0.01,

*represents *P*<0.05, for values versus that obtained in the absence of inhibitor.

### Vardenafil significantly increases the accumulation of intracellular paclitaxel in ABCB1-overexpressing cells by inhibiting drug efflux

**Figure 1:**
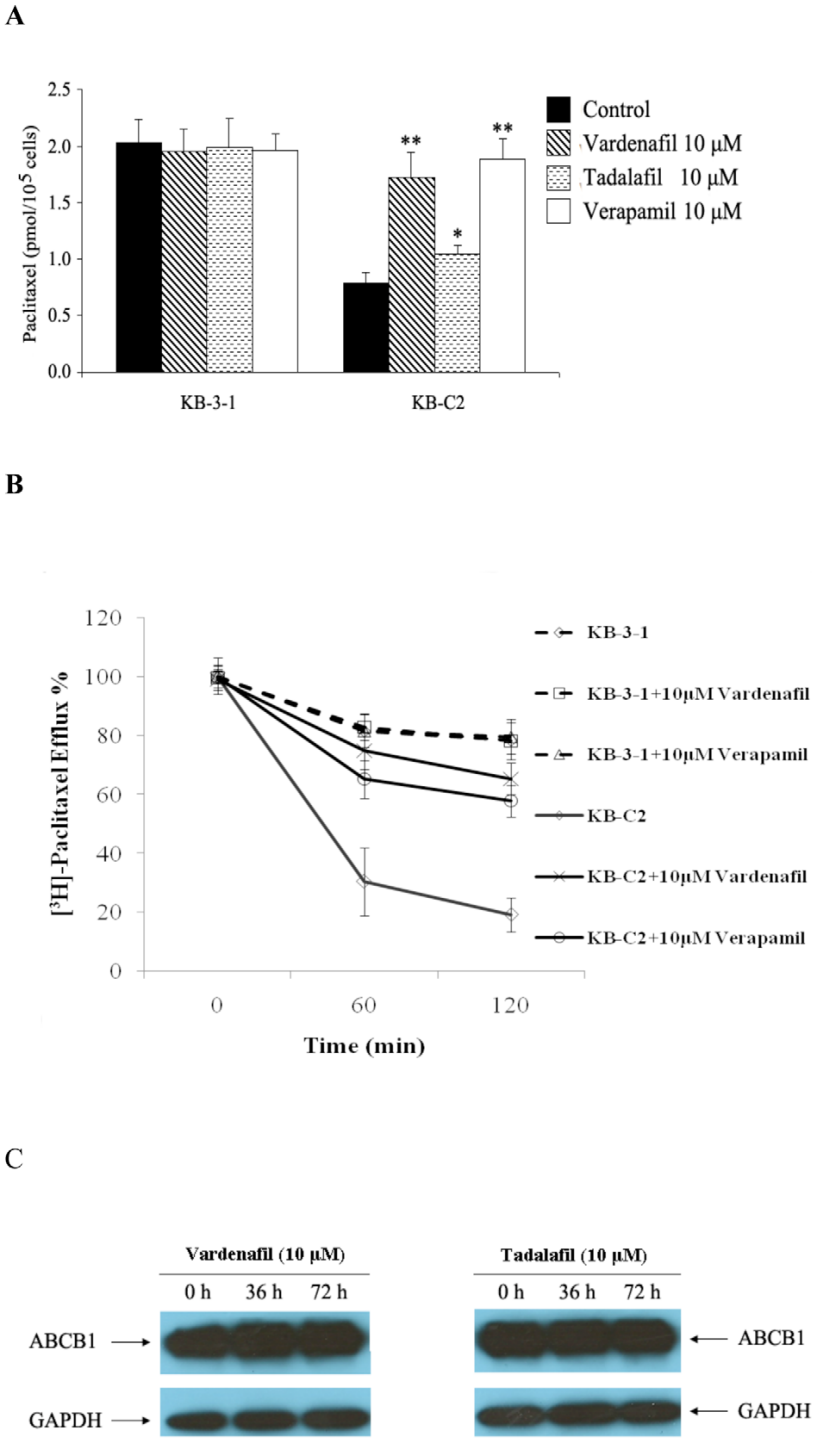
The effect of vardenafil and tadalafil on the accumulation (A), and efflux (B) of [^3^H]-paclitaxel, and ABCB1 expression (C) in ABCB1 overexpressing cells. (**A**) The accumulation of [^3^H]-paclitaxel was measured after cells were pre-incubated with or without vardenifil, tadalafil, or verapamil for 1 h at 37°C and then incubated with 0.1 µM [^3^H]-paclitaxel for another 2 h at 37°C. (**B**) The percentage of the paclitaxel released was plotted as a function of time. After 1 h of incubation of the vardenifil, [^3^H]-paclitaxel was co-incubated in KB-3-1 and KB-C2 cells with or without vardenafil or verapamil. Data points represent the means ± SD of triplicate determinations. * and ** represent *p*<0.05 and *p*<0.01, respectively, for values versus those in the control group. (**C**) Effect of vardenafil (left panel) or tadalafil (right panel) on the expression of ABCB1 for 36 and 72 h, respectively. Independent experiments were performed at least three times, and a representative experiment is shown.

In order to determine vardenafil's reversal mechanism for the ABCB1 transporter, we measured the accumulation of the [**^3^**H]-paclitaxel in the absence and presence of vardenafil. As shown in Figure 1 A, the intracellular concentration of paclitaxel in KB-C2 cells was approximately 55% of that in the parental KB-3-1 cells. However, 10 µM of vardenafil significantly increased the intracellular accumulation of [**^3^**H]-paclitaxel in KB-C2 by 1.6-fold without altering the levels accumulated in KB-3-1 cells (Figure 1 A). In contrast, although tadalafil at 10 µM had significant effect on the paclitaxel accumulation, the accumulation was much less than that of vardenafil and positive control verapamil at 10 µM.

In another series of experiments, we determined the effect of vardenafil on paclitaxel efflux. The intracellular levels of paclitaxel were measured over a period of 2 h (Figure 1B). As expected, a significantly higher concentration of paclitaxel was effluxed from the KB-C2 cells compared to KB-3-1 cells, and the amount of effluxed paclitaxel increased with time. At the one hour time point, 70% of the accumulated paclitaxel was effluxed from the KB-C2 cells in the absence of vardenifil, where as 10 µM of significantly blocked the efflux function of ABCB1, with 75% of the paclitaxel being retained inside the KB-C2 cells (Figure 1B). There was no significant change in the concentration of paclitaxel subjected to efflux in parental KB-3-1 cells in the absence or presence of vardenafil. Thus, vardenifil significantly inhibited paclitaxel efflux from the KB-C2 cells to the extent that efflux from this cell line was comparable to that of the control cells (Figure 1B).

### Vardenafil does not alter the membrane expression of ABCB1

The reversal of ABCB1-mediated MDR could occur by either decreasing ABCB1 expression or by inhibiting ABCB1 activity. To determine the effect of vardenafil on ABCB1 expression, the ABCB1 over-expressing KB-C2 cells were incubated with 10 µM of vardenafil or tadalafil for 36 h and 72 h. The result of the Western blot is given in Figure 1C, and it indicates that the protein level of ABCB1 in KB-C2 cells was not significantly altered after the cells were incubated with vardenafil (left panel) or tadalafil (right panel). Because we used whole cell lysates for the Western blot analysis, it is impossible to determine whether the ABCB1 protein is on membrane or has translocated inside the cell. Therefore, we performed an immunofluorescence assay to see if the location of the ABCB1 transporter was altered. The results suggest neither 10 µM of vardenafil or tadalafil altered the expression of ABCB1 in the membrane of the KB-C2 cells at least after 72 h of incubation (data not shown). Overall, these experiments suggest that neither vardenafil nor tadalafil inhibit the expression of the ABCB1 transporter and they do not alter the translocation of ABCB1 in MDR cancer cells.

### The effect of vardenafil and tadalafil on the ATPase activity of ABCB1

**Figure 2:**
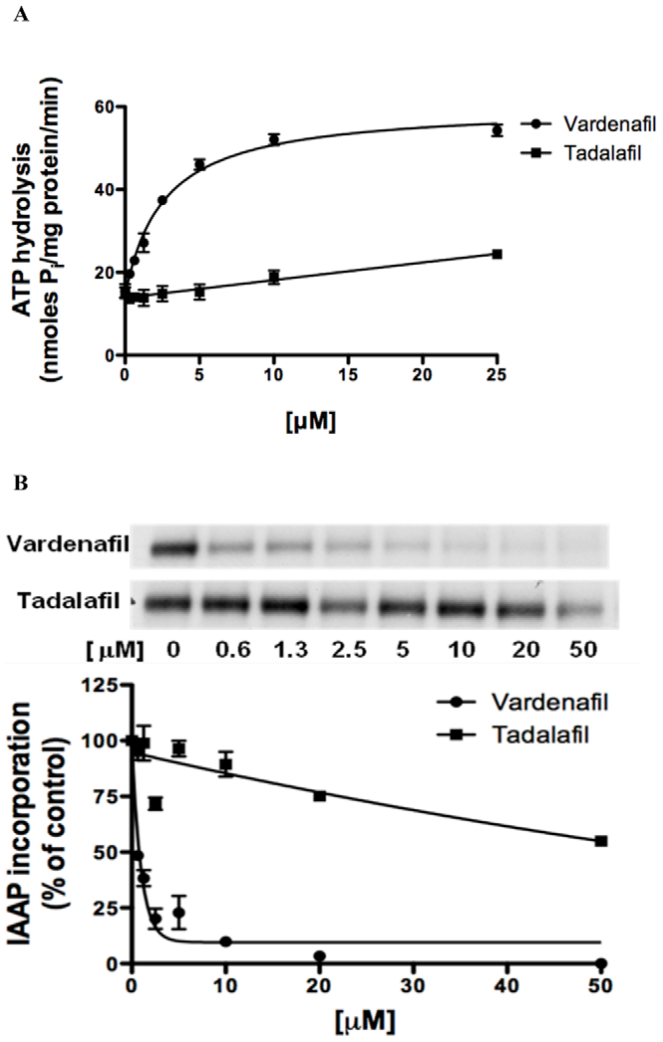
The effect of vardenafil or tadalafil on the ATPase activity of ABCB1 (A) and the photoaffinity labeling of ABCB1 with [^125^I]-IAAP. (B). (**A**) The Vi-sensitive ATPase activity of ABCB1 in membrane vesicles was determined with different concentrations of vardenafil (closed circles) or tadalafil (closed squares) as described previously [24]. The concentration required for 50% stimulation with vardenafil was 2.69±0.72 µM; whereas the stimulation with tadalafil was not saturable. (**B**) The photoaffinity labeling of ABCB1 with [^125^I]-IAAP was performed in the presence or absence of different concentrations of vardenafil (A) or tadalafil (B). The radioactivity incorporated into ABCB1 was determined by exposing the gel to an X-ray film at −70°C. Mean values are given, and the error bars represent standard error from at least three independent experiments.

The drug efflux function of ABCB1 is coupled to ATP hydrolysis by the ATPase enzyme that is usually stimulated in the presence of ABCB1 substrates [28]. To assess the effect of vardenafil and tadalafil on the ATPase activity of ABCB1, the rate of ABCB1-mediated ATP hydrolysis was measured in the isolated membrane vesicles in the presence of various concentrations of vardenafil or tadalafil under conditions that suppressed the activity of the other major membrane ATPases. As shown in Figure 2A, vardenafil produced a concentration-dependent increase in the ATPase activity of ABCB1 over a range of concentrations. The concentration of vardenafil required for 50% stimulation of ATPase activity was 2.69 µM. However, tadalafil only mildly stimulates ATPase activity, without reaching the concentration required for 50% stimulation even at highest concentrations used.

### Effect of vardenafil and tadalafil on the photoaffinity labeling of ABCB1 with [^125^I]-IAAP

In order to determine if vardenifil interacts with the substrate binding sites of ABCB1, we measured the effect of vardenafil and tadalafil on the photoaffinity labeling of the ABCB1 transporter with [^125^I]-IAAP using the membrane vesiclesl. As indicated in Figure 2B, vardenafil inhibited the photoaffinity labeling of ABCB1 with [^125^I]-IAAP in a concentration-dependent manner. Vardenafil, at concentrations of 0.69 µM and 10 µM, inhibited the [^125^I]-IAAP photolabeling of ABCB1 by 50% and 90%, respectively, whereas concentrations of tadalafil up to 50 µM did not produce a 50% inhibition of the [^125^I]-IAAP photolabeling of the ABCB1 transporter.

### Model for binding of vardenafil and tadalafil to ABCB1

**Figure 3:**
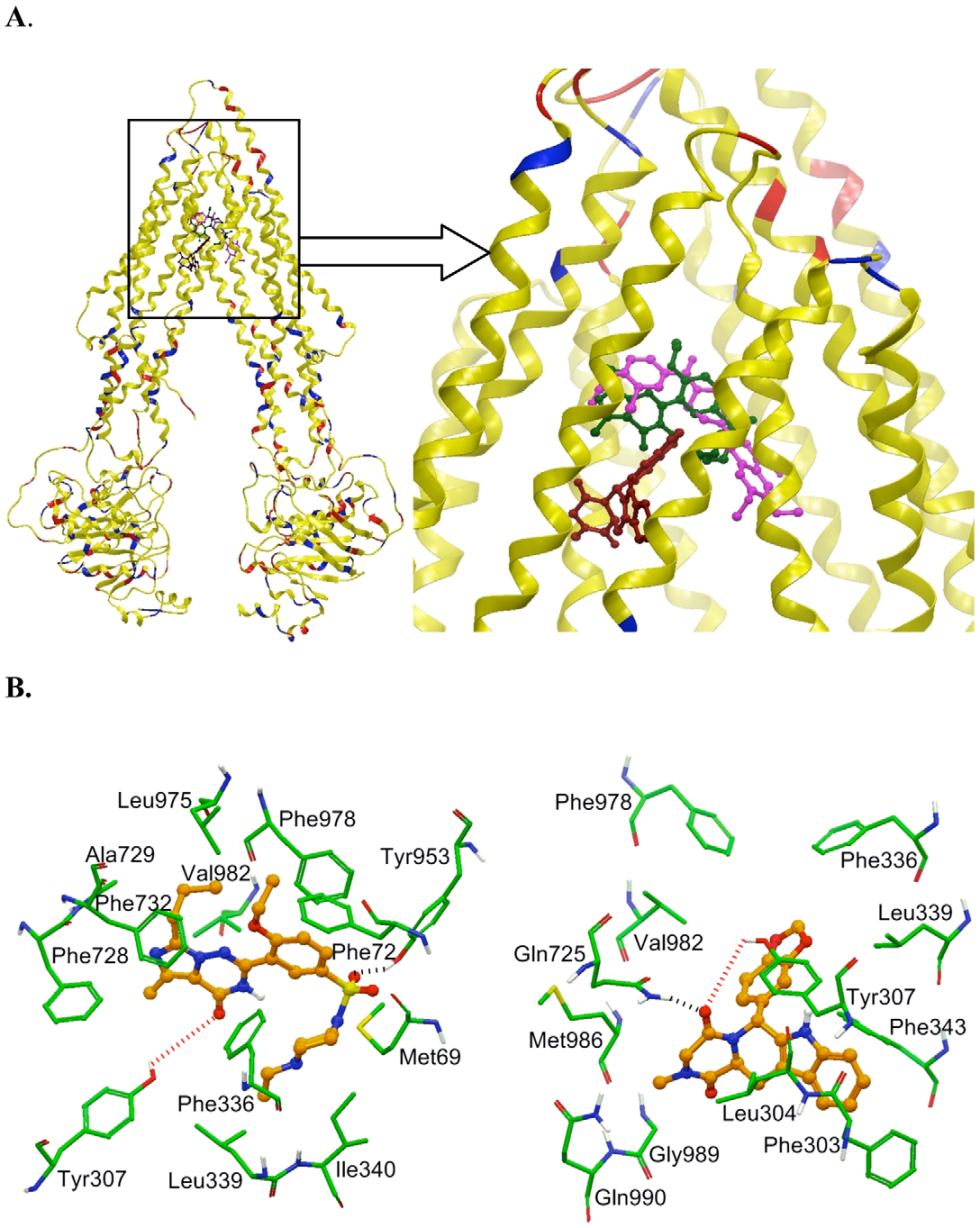
(**A**) The ribbon diagram of open to the cytoplasm-3D structural conformation of a homology model of human ABCB1 based on the crystal structure coordinates of mouse ABCB1. The docked poses of vardenafil (Green), tadalafil (Brown) and IAAP (Purple) as a ball and stick model are shown within the large hydrophobic cavity of ABCB1 at different inhibitor biding sites. (**B**) The XP-Glide predicted binding mode of vardenafil (left panel) and tadalafil (right panel). Important amino acids are depicted as sticks with the atoms colored as carbon – green, hydrogen – white, nitrogen – blue, oxygen – red and sulfur – yellow) whereas the inhibitor is shown as a ball and stick model with the same color scheme as above except carbon atoms are represented in orange. The dotted black line indicates hydrogen bonding interaction whereas a dotted red line indicates interacting distance.

In the present study, the PDE5 inhibitors vardenafil and tadalafil have been described for the first time as ABCB1 inhibitors. Their predicted binding conformation within the large cavity of ABCB1 is yet to be determined. Additionally, the crystal structure of the human ABCB1 remains to be elucidated. Therefore, we sought to develop a homology model of human ABCB1 based on the mouse ABCB1-QZ59-RRR co-crystal structure as a template and use the resulting homology model (Figure 3A) for the Glide docking study of vardenafil and tadalafil. Four binding sites were reported in the crystal structure of mouse ABCB1 as represented by the following sites: ABCB1-QZ59-RRR (site-1), ABCB1-QZ59-SSS (site-2), ABCB1-verapamil (site-3) and the site common to the above three sites (site-4) [27]. Since the photoaffinity labeling data suggested that vardenafil displaces iodoazidoaryl prazosin (IAAP) in a concentration-dependent manner, we also docked IAAP to these sites for comparison. These data also indicated that vardenafil and IAAP share same binding site i.e., site-1, however, the tadalafil binding site is somewhat different from QZ59-RRR site i.e., site-4 as predicted by Glide docking score (Table S1). A comparison of the binding energy data for the docked poses of vardenafil, tadalafil and IAAP at each of the binding sites (Table S1) suggested that most potent ABCB1 inhibitor, vardenafil, exhibited the most favorable binding energy within the QZ59-RRR binding site of ABCB1, whereas tadalafil interacted most favorably with site-4. Thus, the following section will discuss the bound conformation of vardenafil and tadalafil in site-1 and in site-4, respectively. It should be noted that the binding energy data for vardenafil and tadalafil (Table S1) within each of the predicted ABCB1 sites is yet to be experimentally validated. Although the docking results have not been verified by site-directed mutagenesis or co-crystal complex of vardanafil-ABCB1 and tadalafil-ABCB1, in the interim, the binding model of these ABCB1 inhibitors may serve as a guide for further lead optimization studies.

The XP-Glide predicted binding mode of vardenafil indicates the importance of hydrophobic and electrostatic interactions within the large drug binding cavity of ABCB1 (Figure 3B, left panel). While the N-ethylpiperazine (D-ring) of vardenafil forms hydrophobic contacts with Met69, Phe336, Leu339 and Ile340, the C-ring along with ethoxy substituent enters into favorable hydrophobic interactions with Phe72, Leu975 and Phe978. The A-ring, along with its methyl and propyl substituents and the B-ring, are engaged in hydrophobic interactions with the side chains of Phe728, Ala729, Phe732 and Val982. In addition, vardenafil also appeared to form favorable electrostatic interactions with residues Tyr953 and Tyr307. For example, the sulfonyl oxygen atom forms a hydrogen bond with the hydroxyl group of Tyr953 (-SO_2_—HO-Tyr953), whereas the carbonyl function of the B-ring is located at a distance of 4.0 Å from the side chain hydroxyl group of Tyr307.

The XP-Glide predicted binding mode of tadalafil in site-4 of the large drug binding cavity of ABCB1 is shown in Figure 3B (right panel). The A and B-rings of the indole moiety bind to the hydrophobic pocket formed by the side chains of Phe303, Leu304, Tyr307 and Phe343. Moreover, Phe343 is also has hydrophobic contacts with the B-, C- and E-rings. Both E- and F-rings of the benzodioxole moiety are surrounded by the side chains of Phe336, Leu339, Phe978 and Val982. The carbonyl oxygen atom (close to E ring) of the D-ring was stabilized by a hydrogen bonding interaction with the side chain amide group of Gln725 (-CO—H_2_NOC-Gln725). The lower efficacy of tadalafil compared to vardenafil may be due to the orientation of its hydrophobic N-methyl substituent of D-ring towards the unfavorable polar backbone of Met986 and Gly989 and the polar side chain amide group of Gln990.

## Discussion

One of the major mechanisms responsible to MDR in cancer cells is the overexpression of the ABCB1 transporter. [7], [8]. However, currently, none of the ABCB1 inhibitors or modulators have been approved for clinical oncological practice. The present study demonstrates for the first time that vardenifil, a PDE-5 inhibitor used in the treatment of male erectile dysfunction, reverses ABCB1-mediated MDR in a concentration-dependent manner. The magnitude of vardenafil's reversal is similar to that of verapamil, an established, non-selective ABCB1 inhibitor. In addition, it significantly reverses MDR mediated by the ABCB1 transporter in the drug selected cell line KB-C2 to anticancer substrates such as colchicine and paclitaxel, whereas it had no effect on the cytotoxicity to cisplatin, a drug that is not an ABCB1 substrate (Table 1). In order to eliminate the possibility of multiple factors playing a role in drug selected cell lines, we measured the effect of vincristine and paclitaxel cytotoxicity on ABCB1 transfected HEK293/ABCB1 cells (Table 2). Therefore, vardenafil's effect was specific to ABCB1 overexpressing cells but had no significant toxic effects on the parental cells when combined with ABCB1 transporter substrate anticancer drugs. Additionally, vardenifil did not affect the function of other prominent ABC transporters such as ABCC1 and ABCG2 (Table S2) that are widely known to cause MDR. Consistent with the cytotoxicity data, the drug accumulation results (Figure 1A) indicated that vardenafil significantly enhances intracellular paclitaxel accumulation by blocking the efflux of [^3^H]-paclitaxel in KB-C2 cells that overexpress ABCB1 (Figure 1A and 2B). This suggests that vardenafil potentiates the sensitivity of cells to the cytotoxicity of paclitaxel by inhibiting the drug efflux function of ABCB1, thereby increasing the intracellular accumulation of the drug. It is possible that reversal of MDR produced by vardenafil is due to inhibition of its transport function or decreased expression of the ABCB1 transporter protein. The Western blot and immunofluorescence analysis in ABCB1 overexpressing cells incubated with vardenafil or tadalafil indicated that neither drug significantly altered the membrane expression or translocation of the ABCB1 transporter from membrane to intracellular organelles in KB-C2 cells (Figure 1C and data not shown), respectively. These finding are in agreement with our results indicating that vardenafil inhibits ABCB1 function rather than its expression.

In the present study, we also investigated the interaction of vardenafil with the ABCB1 transporter by using the ATPase and photoaffinity labeling ([^125^I]-IAAP) assays. The ATPase activity of the ABC transporters is stimulated in the presence of transport substrates. The substrate-stimulated ATPase activity of ABCB1 is coupled to drug-transport [28]. Since both vardenafil and tadalafil stimulated ABCB1-mediated ATPase activity (Fig. 2 A), these drugs, especially vardenafil might be the transport substrate of ABCB1. The inhibition of IAAP binding by these compounds also demonstrated their interaction at the drug-binding site of ABCB1. In transport assays, vardenafil inhibited the efflux of paclitaxel, which is a substrate of ABCB1 (Fig. 1). We plan to use radiolabeled vardenafil to test whether this drug is transported by ABCB1. In addition, it is important to note that some of the modulators, which are not transported by ABCB1 such as cis-flupentixol and disulfiram, also stimulate ATPase activity of this transporter [29], [30]. The basis for the stimulation of ATPase activity of ABCB1 by modulators is not yet well understood.

As mentioned above, vardenafil is a new PDE-5 inhibitor that is used in the treatment of erectile dysfunction [31]. It competitively inhibits cGMP hydrolysis by PDE-5, thereby increasing cGMP accumulation and relaxation of vascular smooth muscle [32]. The cGMP blocking effect of vardenafil also makes it a promising therapeutic agent for the treatment of pulmonary arterial hypertension, as well as certain cardiovascular dysfunction [33]. Recent data suggest that an increased in PDE-5 expression is linked with the modulation of certain enzymes involved in the proliferation and antiapoptotic mechanisms observed in multiple carcinomas. Thus, the inhibition of PDE-5 may have anticancer effect [34]. For example, Sarfati and colleagues [35] found that vardenafil could induce the caspase-dependent apoptosis in chronic lymphocytic leukemia (CLL) cells. This research group also reported that vardenafil, as well as tadalafil, could reverse tumor-induced immunosuppression [36]. In addition vardenafil has been shown to selectively increase the blood-brain tumor barrier permeability by inhibiting ABCB1, thereby enhancing the effects of chemotherapeutic drugs in a mouse metastatic brain tumor model [37], [38]. The current study demonstrates for the first time that vardenafil significantly reverses MDR mediated by the ABCB1 transporter. We also examined the effect of another PDE-5 inhibitor, tadalafil, on ABCB1-mediated paclitaxel resistance. In contrast to vardenafil, tadalafil, produced only mild reversal of ABCB1 mediated paclitaxel resistance (Table 1 and 2). One possible explanation for this difference may be related to their structures as the molecular structure of vardenafil is markedly different from that of tadalafil (Table S1) [39]. A number of pharmacophore models for ABCB1 inhibitors have identified features such as hydrophobic interactions, hydrogen bond acceptors, aromatic ring center(s) and positive ionizable groups [40]. Thus, ABCB1 preferentially binds to positively charged, amphipathic molecules and this suggests the involvement of acidic residues such as Asp and Glu in drug binding. Although none of the predicted binding sites of the human ABCB1 transporter have acidic residues, there are a few acidic residues located in a region close to the membrane surface and are accessible from within the drug binding sites. These acidic residues are implicated in providing selectivity towards cationic amphipathic drug molecules through ionic interactions during their entry into the drug binding site of ABCB1. In the present study, vardenafil (the most potent ABCB1 inhibitor) exhibit all of the pharmacophoric features (hydrophobic, hydrogen bond acceptor, aromatic ring centers and positive ionizable groups) for interaction with the ABCB1 binding sites, whereas tadalafil (the least potent ABCB1 inhibitor) lacks the positive ionizable group. Although most of the ABCB1 inhibitors block the function of ABCB1 transporter protein by binding to the substrate binding sites, there is evidence for the presence of multiple binding sites [41] and this hinders the development of a conclusive structure-activity relationship for ABCB1 inhibitors. Until the co-crystal structure studies are performed for the vardenafil-ABCB1 complex, the present docking conformation of vardenafil will serve as a guide for further development of imidazotriazinone class of ABCB1 inhibitors.

In summary, this is the first study to indicate that the PDE-5 inhibitor, vardenafil, reverses ABCB1-mediated MDR by directly blocking the drug efflux function of ABCB1 without affecting the expression of the transporter. Based on the data presented here, further *in vivo* studies are warranted to determine if vardenafil can inhibit the ABCB1 transporter and reverse ABCB1-mediated MDR in cancer cells.

## Supporting Information

Table S1 Binding energies of vardenafil, tadalafil and IAAP within each of the predicted binding sites of ABCB1. *^a^*Site represented by bound QZ59-*RRR*. *^b^*Site represented by bound ligand QZ59-*SSS*. *^c^*Verapamil binding site. *^d^*Site grid generated using residues Phe728 and Val 982, which are known to be common to above three sites.
(DOC)

Table S2 The effect of vardenifil and tadalafil on the reversing ABCG2- and ABCC1-mediated drug resistance. Cell survival was determined by MTT assay as described in “Materials and Methods”. Data are the means ± SD of at least three independent experiments performed in triplicate. The fold-reversal of MDR (values given in parentheses) was calculated by dividing the IC_50_ for cells with the anticancer drug in the absence of inhibitor by that obtained in the presence of inhibitor. ** represents *P*<0.01, for values versus that obtained in the absence of inhibitor.
(DOC)
