# Supplementary material for: The Phosphodiesterase-5 Inhibitor Vardenafil Is a Potent Inhibitor of ABCB1/P-Glycoprotein Transporter
Source: PLoS One. 2011 Apr 28;6(4):e19329. doi: 10.1371/journal.pone.0019329 (PMC3084276; doi:10.1371/journal.pone.0019329)
Supplement: Table S1 — Binding energies of vardenafil, tadalafil and IAAP within each of the predicted binding sites of ABCB1. aSite represented by bound QZ59-RRR. bSite represented by bound ligand QZ59-SSS. cVerapamil binding site. dSite grid generated using residues Phe728 and Val 982, which are known to be common to above three sites. (DOC) [file pone.0019329.s001.doc]

**Table S1**.

| **Ligands** | **Glide score kcal/mol** | | | |
| --- | --- | --- | --- | --- |
|  | Site-1^a^ | Site-2^b^ | Site-3^c^ | Site-4^d^ |
| Vardenafil | -8.56 | -6.26 | -5.13 | -4.87 |
| Tadalafil | -6.26 | -5.05 | -7.35 | -7.85 |
| Iodoazidoaryl prazosin (IAAP) | -8.89 | -5.79 | -4.54 | -5.16 |
