# Supplementary material for: The Phosphodiesterase-5 Inhibitor Vardenafil Is a Potent Inhibitor of ABCB1/P-Glycoprotein Transporter
Source: PLoS One. 2011 Apr 28;6(4):e19329. doi: 10.1371/journal.pone.0019329 (PMC3084276; doi:10.1371/journal.pone.0019329)
Supplement: Table S2 — The effect of vardenifil and tadalafil on the reversing ABCG2- and ABCC1-mediated drug resistance. Cell survival was determined by MTT assay as described in “Materials and Methods”. Data are the means ± SD of at least three independent experiments performed in triplicate. The fold-reversal of MDR (values given in parentheses) was calculated by dividing the IC50 for cells with the anticancer drug in the absence of inhibitor by that obtained in the presence of inhibitor. ** represents P<0.01, for values versus that obtained in the absence of inhibitor. (DOC) [file pone.0019329.s002.doc]

**Table S2.**

| **Compounds** | **IC50 ± SD (μM) (fold reversal)** | |
| --- | --- | --- |
|  | HEK293/pcDNA3.1 | ABCG2-482-R2 (ABCG2) |
| SN-38 | 0.1157 ± 0.0151 (1.00) | 3.6471 ± 0.5472 (1.00) |
| + Vardenafil 10 μM | 0.1049 ± 0.0186 (1.10) | 3.7652 ± 0.6264 (0.97) |
| + Tadalafil 10 μM | 0.1202 ± 0.0250 (0.96) | 3.5173 ± 0.8198 (1.04) |
| + FTC 10 μM | 0.1021 ± 0.0238 (1.13) | 0.9484 ± 0.1634** (3.84) |
|  |  |  |
|  | HEK293/pcDNA3.1 | HEK/MRP1 (ABCC1) |
| Vincristine | 0.0012 ± 0.0002 (1.00) | 0.0106 ± 0.0022 (1.00) |
| + Vardenafil 10 μM | 0.0013 ± 0.0001 (0.92) | 0.0099 ± 0.0016 (1.07) |
| + Tadalafil 10 μM | 0.0011 ± 0.0002 (1.09) | 0.0103 ± 0.0021 (1.03) |
| + ONO-1078 10 μM | 0.0009 ± 0.0001 (1.33) | 0.0018 ± 0.0003** (5.9) |
|  | | |
